# Targeting magnesium homeostasis: a novel therapeutic strategy for liver diseases

**DOI:** 10.3389/fnut.2026.1709477

**Published:** 2026-03-04

**Authors:** Lili Ji, Hanhan Yu, Ruwen Wang, Hongmei Yan, Xiaofeng Yin, Shanshan Guo, Ru Wang

**Affiliations:** 1 School of Exercise and Health, Shanghai University of Sport, Shanghai, China; 2 Department of Endocrinology and Metabolism, Zhongshan Hospital, Fudan University, Shanghai, China; 3 Research Department of Talent Identification and Development in Youth Sports, Shanghai Research Institute of Sports Science (Shanghai Anti-Doping Agency), Shanghai, China

**Keywords:** ALD, DILI, HCC, hypomagnesemia, magnesium homeostasis, MASLD, therapeutic strategy

## Abstract

This review systematically examines a novel therapeutic strategy for liver disease prevention and treatment by targeting magnesium ion homeostasis. Magnesium ions (Mg^2+^), an essential macromineral, plays a critical role in energy metabolism and enzymatic activity, with its systemic balance maintained through intestinal absorption, renal excretion, and skeletal storage. Emerging evidence demonstrates that hypomagnesemia or intracellular magnesium deficiency is strongly associated with the development and progression of various liver diseases, including metabolic dysfunction-associated steatotic liver disease (MASLD), alcoholic liver disease (ALD), drug-induced liver injury (DILI), and hepatocellular carcinoma (HCC). Mechanistically, magnesium deficiency exacerbates hepatic pathology by promoting insulin resistance, impairing mitochondrial function, inducing oxidative stress and inflammatory responses, disrupting gut–liver axis homeostasis, and compromising DNA repair and anti-tumor immunity. Preclinical and preliminary clinical studies indicate that restoring magnesium homeostasis—through dietary supplementation, magnesium-based pharmacological agents, or modulation of magnesium transporters [e.g., inhibition of the Mg^2+^ efflux transporter Cyclin M4 (CNNM4)]—can improve metabolic function in hepatocytes, attenuate inflammation and fibrosis, and exert hepatoprotective effects. Collectively, these findings highlight magnesium homeostasis as a promising therapeutic target for liver disease, warranting further validation in large-scale clinical trials to facilitate clinical translation.

## 1 Introduction

Liver diseases constitute a major and growing global health challenge, responsible for over 2 million deaths annually—approximately 4% of worldwide mortality (1). While viral hepatitis remains a significant burden, the rising prevalence of MASLD and ALD is driving a concerning upward trajectory in liver-related mortality, a trend projected to intensify in coming decades (1, 2). This escalating burden underscores an urgent, unmet need for innovative therapeutic and preventive strategies.

In this context, the regulation of Mg^2+^ homeostasis has emerged as an area of growing translational interest in hepatology. Mg^2+^ is an essential cofactor for hundreds of enzymatic reactions and is indispensable for fundamental cellular processes, including energy metabolism and genomic stability (3). Accumulating epidemiological evidence links hypomagnesemia and low dietary magnesium intake to an increased risk and severity of major liver diseases, including MASLD, ALD, and HCC (4–6). Notably, data from the NHANES III cohort demonstrate that each 100 mg/day increase in magnesium intake is associated with a 49% reduction in liver-related mortality (7), underscoring the potential clinical relevance of magnesium status.

Importantly, these associations are best interpreted within a bidirectional pathophysiological framework. Liver disease itself can disrupt systemic and cellular magnesium homeostasis, while the resulting magnesium deficiency may subsequently act as a disease-modifying amplifier, exacerbating hepatic injury through mechanisms such as chronic inflammation, mitochondrial dysfunction, and oxidative stress (8, 9). This reciprocal relationship positions magnesium dysregulation not as a primary etiological driver, but as a modifiable factor that may influence disease progression and outcomes.

Despite this compelling biological rationale, significant translational gaps persist. The precise mechanisms, particularly those involving hepatocellular magnesium transporters, remain incompletely defined. Moreover, the clinical efficacy of correcting magnesium imbalance—whether through dietary supplementation or targeted interventions—is inconsistent and hampered by a lack of biomarker-driven, precision approaches.

This review aims to provide a critical synthesis and framework to bridge these gaps. We will first delineate the systemic and cellular principles of magnesium homeostasis. We will then systematically evaluate the bidirectional evidence linking its dysregulation to the pathogenesis of major liver diseases (MASLD, ALD, DILI, and HCC), with dedicated focus on emerging mechanistic players such as the magnesium efflux transporter CNNM4. Finally, we will map the therapeutic landscape—from nutrient repletion to transporter-targeted strategies—organizing them within a hierarchy of translational readiness and delineating key scientific and clinical challenges. By integrating nutritional, molecular, and clinical perspectives, this review positions the strategic modulation of magnesium homeostasis as a mechanism-informed axis for potential adjunctive intervention in the comprehensive management of liver disease.

## 2 Methodology

This narrative review was conducted to comprehensively synthesize contemporary evidence on the role of magnesium homeostasis dysregulation in the pathogenesis and treatment of liver diseases. To ensure a systematic and transparent literature acquisition process, a standardized search strategy was applied across major academic databases, including PubMed, Embase, Web of Science Core Collection, and Cochrane Library.

The literature search utilized a combination of key terms and Boolean operators (AND/OR) to capture studies relevant to the core themes of this review. The search strategy primarily revolved around the following conceptual groups: Magnesium Status and Liver Disease: Terms such as “hypomagnesemia,” “magnesium deficiency,” and “magnesium homeostasis” were combined with general (“liver disease,” “hepatopathy”) and specific liver disease terms (“metabolic dysfunction-associated steatotic liver disease (MASLD),” “metabolic dysfunction-associated steatohepatitis (MASH),” “alcoholic liver disease (ALD),” “drug-induced liver injury (DILI),” “hepatocellular carcinoma (HCC),” “liver cirrhosis”). Pathogenic Mechanisms: “Magnesium” was paired with mechanism-related terms like “insulin resistance,” “mitochondrial dysfunction,” “oxidative stress,” “inflammation,” and “gut-liver axis.” Therapeutic Strategies: Searches included terms for interventions (“magnesium supplementation,” “magnesium isoglycyrrhizinate,” “Sodium-glucose cotransporter 2 (SGLT2) inhibitors,” “inulin fibers”) and molecular targets (“magnesium transporters,” “Cyclin M4 (CNNM4),” “transient receptor potential melastatin 6 and 7 (TRPM6/7),” “solute carrier family 41 member 1 (SLC41A1)”).

Titles, keywords, and abstracts of retrieved records were initially screened for relevance to the review’s focus. Full-text articles were then assessed for inclusion based on the following criteria: (a) peer-reviewed original research or review articles; (b) publication date up to September 1, 2025; (c) content providing mechanistic insights, clinical evidence (e.g., epidemiological associations, serum magnesium levels), or preclinical data (e.g., from animal or cell models) directly linking magnesium homeostasis to liver disease etiology, progression, or treatment. Non-English articles, conference abstracts, editorials, and studies not primarily focused on liver diseases were excluded.

Eligible literature was critically evaluated, with the strength of evidence considered based on study design, sample size, and consistency with existing literature. Key findings were then synthesized narratively to construct the current review, integrating nutritional, molecular, and clinical perspectives on targeting magnesium homeostasis in hepatology.

## 3 Regulation of magnesium homeostasis

### 3.1 Cellular Mg^2+^ homeostasis

Approximately 99% of total body magnesium is localized within cells, either in free or protein-bound form, making Mg^2+^ the second most abundant intracellular cation after potassium (3, 10). To preserve this critical ion reservoir, cells employ a sophisticated regulatory system composed of multiple Mg^2+^ channels and transporters—including TRPM6/7, magnesium transporter 1 (MagT1), solute carrier family 41 members 1–3 (SLC41A1–3), mitochondrial RNA splicing 2 (MRS2), and transmembrane protein 94 (TMEM94)—that collectively maintain dynamic equilibrium across the cytoplasm and organelles. The specific mechanisms underlying this process have been elaborated in multiple systematic reviews (10, 11).

Recent studies have highlighted a pivotal role for the hepatocellular Mg^2+^ transporter CNNM4 in liver pathology. Studies have demonstrated that CNNM4 expression is markedly upregulated in liver tissues from patients and experimental models of MASH (8), DILI (6), and ALD (12), with expression levels correlating closely with disease severity. Mechanistic investigations revealed that CNNM4 overexpression enhances Mg^2+^ efflux from the cytoplasm and organelles (including mitochondria and the endoplasmic reticulum), thereby disrupting intracellular magnesium homeostasis. This imbalance compromises mitochondrial activity, aggravates endoplasmic reticulum stress, and accelerates disease progression. Importantly, liver-specific silencing of Cnnm4 in disease models effectively restored intracellular magnesium levels, improved mitochondrial function, attenuated endoplasmic reticulum stress, and significantly delayed disease progression.

Collectively, CNNM4 may play an important role in hepatocellular magnesium dysregulation. While its inhibition represents a potentially promising therapeutic avenue, current evidence is largely derived from a limited number of preclinical models, and human causal validation remains unavailable.

### 3.2 Systemic regulation of body Mg^2+^ homeostasis: the gut-kidney-bone axis

**Figure 1:**
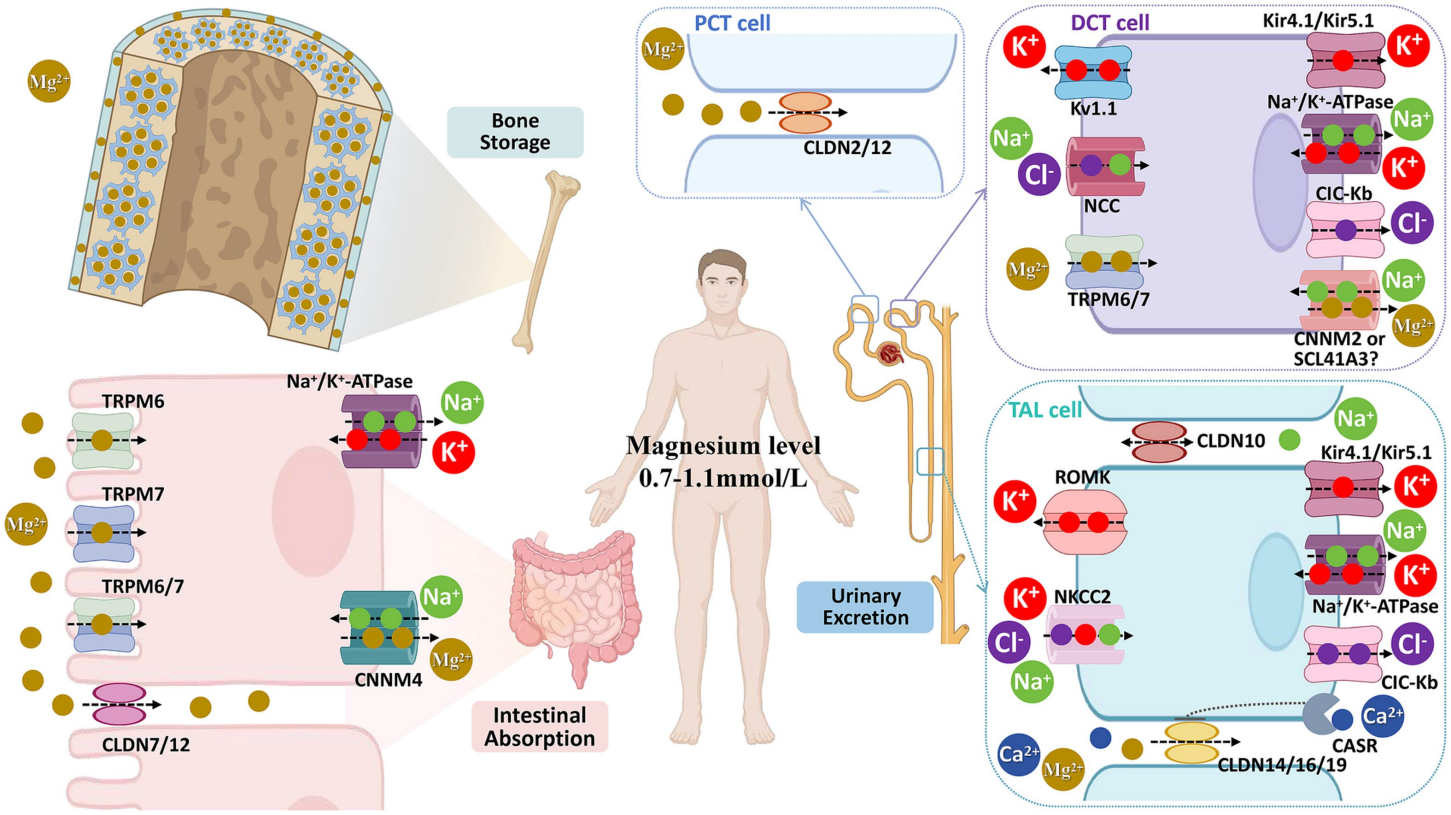
Magnesium ion (Mg^2+^) homeostasis in the body is primarily maintained by three key systems: the intestine (regulating dietary magnesium absorption), the bone (storing magnesium in the form of hydroxyapatite), and the kidneys (regulating urinary magnesium excretion). TRPM6, transient receptor potential melastatin type 6; TRPM7, transient receptor potential melastatin type 7; CLDN7/12, claudin-7/12; Na+/K + -ATPase; CNNM4, cyclin M4; CLDN2/12, claudin-2/12; Kv1.1, voltage-gated K + channel 1.1; NCC, Na + -Cl − cotransporter; Kir, inward rectifier-type K + channel; CIC-Kb, chloride channel Kb; CNNM2, cyclin M2; SCL41A3, solute carrier family 41 member 3; CLDN10, claudin-10; ROMK, renal outer medulla K + channel; NKCC2, Na + -K + -2Cl − cotransporter; CLDN14/16/19, claudin-4/16/19; CIC-Kb, voltage-gated Cl − channel Kb; PCT, proximal convoluted tubule; DCT, distal convoluted tubule; TAL, thick ascending limb.

Systemic magnesium homeostasis is maintained through the coordinated interplay of the intestine, kidneys, and bone—collectively termed the gut–kidney–bone axis—which regulates absorption, excretion, and storage, respectively (Figure 1). This integrative framework highlights how disturbances at any point (e.g., impaired intestinal absorption, excessive renal loss, or depleted bone stores) can disrupt whole-body magnesium balance. Thus, Figure 1 provides an organ-level physiological framework for understanding the systemic origins of hypomagnesemia frequently observed in liver disease, setting the stage for the disease-specific mechanisms discussed below.

#### 3.2.1 Intestinal magnesium absorption

Dietary magnesium is the sole exogenous source of systemic magnesium, making efficient intestinal absorption essential. Absorption occurs primarily in the small intestine, with the colon providing a smaller contribution (3, 13). Two pathways mediate intestinal magnesium uptake: (1) the transcellular pathway, in which Mg^2+^ enters enterocytes through TRPM6/7 channels and exits into the circulation via CNNM4-mediated secondary active transport; and (2) the paracellular pathway, in which Mg^2+^ diffuses passively through tight junctions formed by Claudin-7 and Claudin-12 (13, 14). Of these, the paracellular route accounts for the majority of small intestinal absorption (3). Absorptive efficiency is inversely related to intake level—ranging from 65% at low intakes to 11% at high intakes (15). Multiple factors further modulate absorption, including hormones (e.g., 25-hydroxyvitamin D, parathyroid hormone, fibroblast growth factor-23), luminal acidity, pH-sensitive channels, proton pump inhibitors, and the gut microbiota (14). Under pathological conditions such as diarrhea, magnesium may also be secreted into the intestinal lumen along with water and electrolytes (16).

#### 3.2.2 Renal magnesium reabsorption and excretion

As the “gatekeeper” of magnesium homeostasis, the kidneys maintain systemic magnesium balance through precise regulation of magnesium reabsorption and excretion. Approximately 2,400 mg of magnesium ions (Mg^2+^) are filtered by the glomerulus daily, with 95–99% reabsorbed along the nephron, leaving only ~100 mg excreted in urine. This reabsorption is distributed across three nephron segments with distinct contributions: the proximal convoluted tubule (5–15% of filtered load), the thick ascending limb (TAL) of the loop of Henle (70–80%, the primary reabsorptive site), and the distal convoluted tubule (DCT) (5–10%).

Although the contribution of the DCT to overall reabsorption is relatively low, it plays a pivotal role in determining final urinary magnesium excretion, accounting for 70–80% of the magnesium delivered from the loop of Henle (17). Reabsorption mechanisms vary across nephron segments: in the proximal tubule, magnesium is mainly reabsorbed via a paracellular pathway dependent on Claudin-2/12 proteins, driven by chemical gradients generated by sodium-dependent water reabsorption, which creates a lumen-positive potential (18); in the TAL, magnesium is transported through paracellular channels formed by Claudin-14, Claudin-16, and Claudin-19, which are regulated by a lumen-positive transepithelial voltage gradient (3, 19); in the DCT, reabsorption occurs via a transcellular mechanism involving TRPM6/TRPM7 heteromers (3, 20).

#### 3.2.3 The skeletal magnesium reservoir

Bone serves as the primary reservoir of Mg^2+^, accounting for over 50% of the body’s total magnesium stores (16). Magnesium in bone exists in a functional dual-pool distribution: approximately 30% resides at the surface of bone minerals (surface-exchangeable pool), which contributes to short-term regulation of plasma magnesium levels through rapid exchange; the remaining 70% is embedded within the bone mineral lattice (structural pool), forming the foundation of the bone matrix (21, 22). Consequently, bone—especially the surface-exchangeable magnesium pool—functions as a critical dynamic buffer, playing a key role in the maintenance of systemic magnesium homeostasis. Notably, magnesium content in bone declines with age, from 50% in adolescence to 33% in adulthood, and to approximately 10% in older age (23). This age-related reduction in bone magnesium may impair long-term magnesium balance, contributing to dysregulated systemic magnesium homeostasis.

## 4 Magnesium deficiency and its role in liver diseases

Magnesium deficiency is closely associated with the onset and progression of various liver diseases. In adults, hypomagnesemia is defined as a serum magnesium concentration below the normal range of 0.7–1.0 mmol/L (21, 24). This condition, first documented in the 1850s (25), can result from multiple causes including alcohol abuse, certain medications, malnutrition, gastrointestinal disorders, and respiratory alkalosis (26, 27). Its recognition as a nutrient of public health concern by the U. S. Dietary Guidelines Advisory Committee in 2015 underscores its clinical relevance (28). A key diagnostic challenge is that serum levels may not accurately reflect total body magnesium stores, as tissue depletion can occur covertly (29)—a critical consideration in managing liver disease.

Accumulating evidence indicates that magnesium deficiency is prevalent in patients with liver diseases and likely exacerbates metabolic dysfunction, inflammation, and cellular injury. However, the relationship is not merely unidirectional. Current data increasingly support a bidirectional model: underlying liver disease can disrupt magnesium homeostasis through impaired intestinal absorption, altered storage, and renal wasting (23, 30, 31), while the resulting magnesium deficiency subsequently acts as a disease modifier, amplifying pathological processes such as oxidative stress and inflammatory signaling.

**Figure 2:**
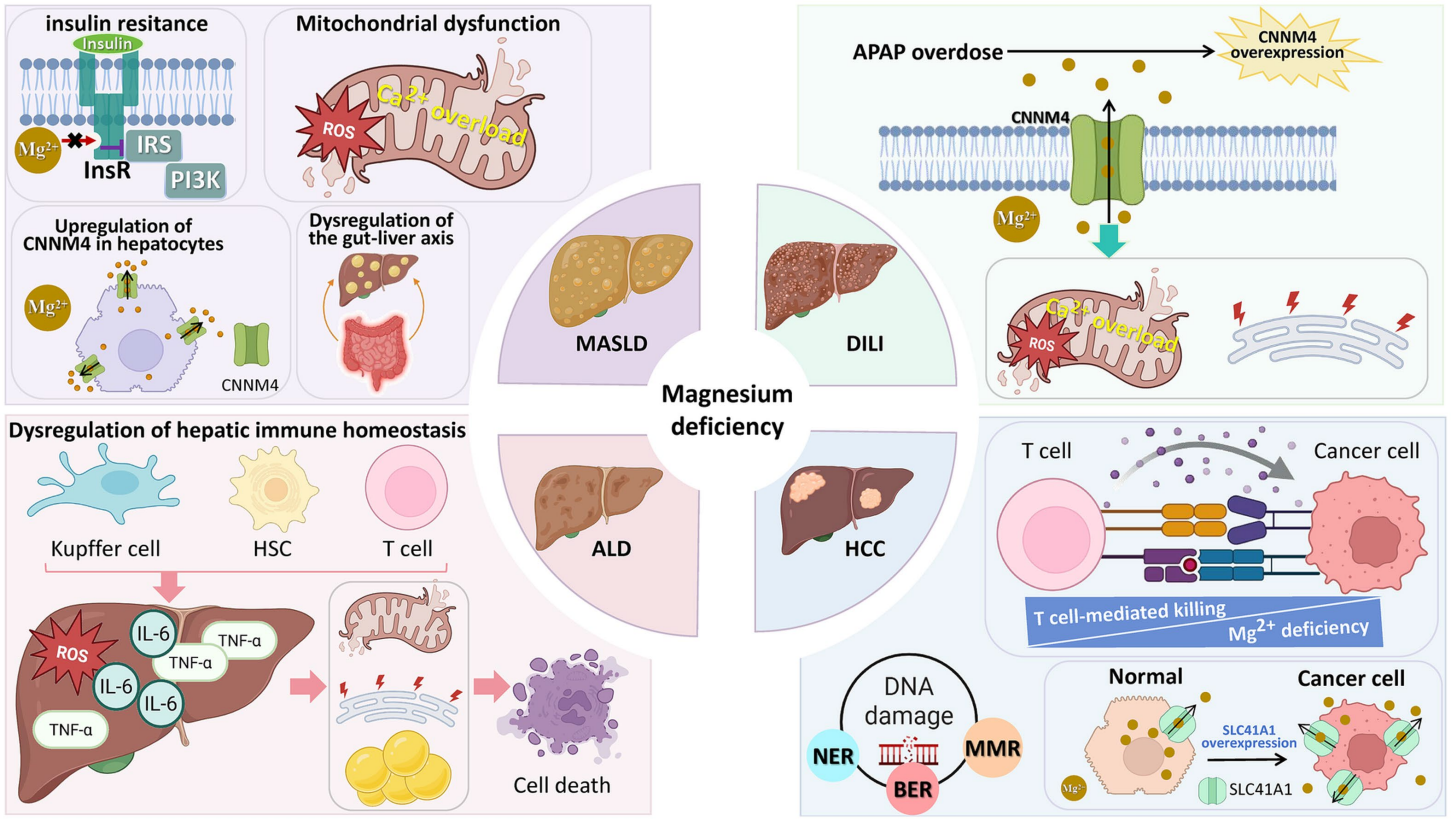
The contribution of magnesium deficiency to multiple liver diseases. Magnesium deficiency exerts pathogenic effects through distinct mechanisms across various liver conditions, including MASLD, ALD, DILI, and HCC. MASLD, metabolic dysfunction-associated steatotic liver disease; ALD, alcoholic liver disease; DILI, drug-induced liver injury; HCC, hepatocellular carcinoma; InsR, insulin receptor; IRS, insulin receptor substrate; PI3K, phosphoinositide 3-kinase; ROS, reactive oxygen species; CNNM4, cyclin M4; HSC, hepatic stellate cell; IL-6, interleukin-6; TNF-*α*, tumor necrosis factor-α; NER, nucleotide excision repair; BER, base excision repair; MMR, mismatch repair; SLC41A1, solute carrier family 41 member 1; APAP, acetaminophen.

The following sections first outline the shared mechanistic pathways through which magnesium dysregulation contributes to liver injury, providing a conceptual foundation. This is followed by a detailed analysis of the epidemiological links, disease-specific mechanisms, and therapeutic implications for MASLD, ALD, DILI, and HCC. Figure 2 visually integrates this framework, illustrating both the common pathways (e.g., mitochondrial dysfunction, gut–liver axis disruption) and their disease-specific manifestations, while emphasizing the bidirectional interplay between liver pathology and magnesium homeostasis. This schematic serves as a roadmap for the mechanistic discussions that follow.

### 4.1 Shared pathogenic mechanisms of magnesium deficiency in liver injury

Magnesium deficiency contributes to liver injury through several convergent biological pathways that are observed across diverse etiologies. These shared mechanisms include the amplification of oxidative stress, impairent of mitochondrial energy metabolism, dysregulation of calcium–magnesium homeostasis, activation of inflammatory signaling cascades, and promotion of fibrogenesis. Importantly, the mechanistic insights summarized in this section are derived predominantly from animal models and *in vitro* systems. Direct causal evidence from large-scale human interventional studies remains limited, and therefore these mechanisms should be interpreted as biologically plausible but largely hypothesis-generating.

Experimental studies consistently demonstrate that magnesium deficiency enhances neutrophil activation and reactive oxygen species (ROS) production while suppressing antioxidant defense systems, thereby promoting lipid peroxidation and oxidative hepatocellular injury (32, 33). At the mitochondrial level, magnesium serves as an essential cofactor for enzymes involved in oxidative phosphorylation and intermediary metabolism. Magnesium depletion has been shown to disrupt the NADH/NAD^+^ redox balance, impair fatty acid *β*-oxidation, and reduce ATP synthesis, collectively compromising hepatocellular energy homeostasis (34).

Hypomagnesemia disrupts cellular calcium–magnesium homeostasis, often leading to relative calcium overload. Mg^2+^ serves as an endogenous calcium channel blocker. Consequently, Mg^2+^ deficiency reduces the inhibitory effect on the mitochondrial calcium uniporter (MCU), thus promoting mitochondrial calcium overload (35). This calcium overload promotes the opening of the mitochondrial permeability transition pore (mPTP), a key event triggering hepatocyte death—effects that are antagonized by Mg^2+^ in experimental systems (36, 37). Consequently, magnesium deficiency-induced mitochondrial calcium dysregulation contributes to oxidative stress, impaired ATP production, and ultimately, hepatocellular injury.

Beyond oxidative and metabolic stress, magnesium deficiency has also been linked to exaggerated hepatic inflammatory responses. Reduced intracellular magnesium enhances Toll-like receptor 4 (TLR4) signaling in Kupffer cells, leading to increased production of pro-inflammatory cytokines such as TNF-*α* and IL-6 (34, 38, 39). Sustained inflammatory activation can, in turn, promote hepatic stellate cell (HSC) activation and extracellular matrix deposition, thereby contributing to fibrogenesis. Although animal studies suggest that magnesium supplementation can attenuate experimental liver fibrosis (40–42), these findings have not yet been substantiated by robust clinical trials in humans.

Collectively, these shared mechanisms support a conceptual framework in which magnesium deficiency acts as a disease-modifying factor that amplifies oxidative stress, mitochondrial vulnerability, inflammation, and fibrogenic signaling across liver disease etiologies. Nevertheless, the relative contribution of magnesium dysregulation to liver injury likely varies by disease context and stage, underscoring the need for cautious interpretation and disease-specific analysis, as discussed in the following sections.

### 4.2 Magnesium deficiency in MASLD

#### 4.2.1 Epidemiological and clinical associations

MASLD is the most common chronic liver disorder worldwide, affecting approximately 38% of adults and 7–14% of children and adolescents, with adult prevalence projected to exceed 55% by 2040 (43, 44). Beyond hepatic manifestations, MASLD is associated with increased all-cause mortality and multiple systemic comorbidities (45). Observational cohort studies consistently report an association between low serum magnesium levels and both the prevalence and severity of MASLD (4, 5, 46, 47). However, these epidemiological findings demonstrate association, not causation, and may reflect reverse or bidirectional relationships within the complex pathophysiology of metabolic syndrome.

#### 4.2.2 MASLD-specific pathogenic mechanisms

Beyond the shared pathways of oxidative stress and inflammation, magnesium deficiency interacts with MASLD-specific pathophysiology in several key areas, predominantly through bidirectional relationships.

A complex, bidirectional relationship with insulin resistance (IR) is central. IR can promote renal magnesium wasting (34), while magnesium deficiency may impair insulin receptor signaling (48, 49), potentially creating a vicious cycle. The temporal hierarchy between these processes remains unresolved.

Regarding mitochondrial dysfunction, a hallmark of MASLD, the role of magnesium deficiency requires careful contextualization. Mitochondrial injury in MASLD is primarily driven by overnutrition, lipotoxicity, and IR (43). Within this multifactorial setting, magnesium depletion is unlikely to be a primary cause but may act as a disease modulator that lowers the cellular threshold for dysfunction and amplifies injury.

The link with gut-liver axis dysregulation is an area of active research. Preclinical models suggest a pathway whereby magnesium deficiency impairs intestinal barrier function, promoting translocation of microbial products like lipopolysaccharide (LPS) and activating hepatic inflammation (50, 51). Magnesium repletion can improve barrier integrity and attenuate injury in these models (51–53). However, the causal sequence in humans is unclear and likely bidirectional. A parsimonious interpretation is that the MASLD milieu (e.g., metabolic inflammation, diet) can independently induce both magnesium deficiency and gut dysbiosis, which may mutually reinforce each other and contribute to disease progression.

#### 4.2.3 Therapeutic interventions and limitations

Recent preclinical studies posit that dysregulation of hepatocellular Mg^2+^ transport, particularly via the efflux transporter CNNM4, may be a modifiable node in experimental MASLD/MASH. In mouse models, hepatic CNNM4 is upregulated, and its liver-specific knockdown increases intracellular Mg^2+^, alleviates endoplasmic reticulum stress, and promotes lipid export via enhanced microsomal triglyceride transfer protein (MTTP) activity and very low density lipoprotein (VLDL) secretion, thereby reducing steatosis (8).

However, the translational relevance of targeting CNNM4 remains highly uncertain. The evidence is confined to a limited set of animal studies, with no human genetic or interventional data to establish a causal role in MASLD. Critical questions regarding the feasibility, safety, and specificity of hepatocyte-targeted CNNM4 inhibition in humans are entirely unresolved.

Therefore, CNNM4 should currently be regarded as an exploratory, hypothesis-generating target, not a clinical strategy. More broadly, these findings reinforce the concept that altered hepatocellular Mg^2+^ handling may modulate disease severity within a bidirectional framework. Future translation will require studies integrating human genetics, direct tissue magnesium assessment, and rigorous interventional trials.

### 4.3 Magnesium deficiency in ALD

#### 4.3.1 Epidemiological and clinical associations

ALD accounts for approximately 60–80% of liver-related deaths in Europe and remains a leading indication for liver transplantation in Western countries (54). Observational studies consistently associate hypomagnesemia with markers of ALD severity and increased mortality risk (55–57), although these data do not establish causality. A limited number of randomized, double-blind, placebo-controlled trials have investigated magnesium supplementation. Evidence from RCTs of limited size and duration has reported that short-term (6–8 weeks) oral magnesium supplementation reduces serum aminotransferase levels, particularly aspartate aminotransferase (AST), in individuals with chronic alcohol use (58, 59).

However, these trials are characterized by relatively small cohorts, short intervention periods, and a reliance on surrogate biochemical endpoints rather than hard clinical outcomes such as disease progression or mortality.

Therefore, while current evidence suggests an association between magnesium deficiency and ALD severity, and indicates possible short-term biochemical benefits from supplementation, the overall clinical evidence remains preliminary. Definitive conclusions regarding therapeutic efficacy, optimal dosing, and long-term safety will require larger, longer-term randomized trials with clinically meaningful endpoints.

#### 4.3.2 ALD-specific pathogenic mechanisms

In ALD, magnesium dysregulation is uniquely positioned as both a consequence and amplifier of injury. Chronic alcohol exposure is a potent direct cause of systemic magnesium deficiency, primarily through two immediate mechanisms: (1) dose-dependent impairment of intestinal absorption via mucosal damage and transporter (e.g., TRPM6/7) inhibition (30), and (2) a rapid, profound increase in renal excretion, with losses rising up to 167% within minutes of intake and persisting despite hypomagnesemia (9, 31). This creates a sustained negative balance, exacerbated by ongoing consumption (60).

This systemic deficit drives hepatocellular magnesium depletion. Experimental data show alcohol induces a time- and dose-dependent efflux of intracellular Mg^2+^, reducing content by 5–25% and impairing ATP production (61, 62), a process linked to ethanol’s disruption of protein kinase Cε-mediated transport (63).

The resulting intracellular deficiency then lowers the threshold for alcohol’s inherent toxicity. Preclinical models indicate that a low-magnesium state sensitizes the liver by potentiating key alcohol-injury pathways: enhancing TLR4 signaling and ROS generation to amplify inflammation (34, 38, 39), and potentially through upregulation of the magnesium efflux transporter CNNM4 (55). Thus, alcohol initiates magnesium loss, which in turn exacerbates the metabolic and inflammatory stress caused by alcohol itself—a classic disease-modifying cycle. Human validation of this cascade, particularly the therapeutic relevance of CNNM4, remains outstanding.

Thus, magnesium deficiency, initiated by alcohol itself, functions as a key disease-modifying amplifier within the pathogenic cascade of ALD. Full human validation of this cycle, including the therapeutic relevance of targets like CNNM4, remains outstanding.

#### 4.3.3 Therapeutic interventions and limitations

Experimental and early clinical studies suggest that restoring magnesium homeostasis may attenuate alcohol-associated inflammatory responses. For instance, magnesium supplementation has been shown to reduce endotoxin-induced systemic inflammation in experimental models (64), and magnesium sulfate can inhibit macrophage activation via blockade of L-type calcium channels (65). Additionally, magnesium isoglycyrrhizinate (MgIG) may alleviate oxidative injury in models of alcoholic hepatitis by suppressing neutrophil activation and infiltration (66).

Nevertheless, evidence supporting therapeutic efficacy in humans remains preliminary. Existing intervention trials are limited by short duration, small sample sizes, heterogeneity in formulations and dosing, and a reliance on surrogate biochemical endpoints rather than hard clinical outcomes (58, 59). The impact of magnesium supplementation on long-term outcomes such as fibrosis progression, cirrhosis, or mortality remains unclear.

Consequently, while magnesium repletion represents a biologically plausible adjunctive strategy, its clinical utility in ALD requires validation in rigorously designed, long-term randomized controlled trials.

### 4.4 Magnesium deficiency in DILI

#### 4.4.1 Epidemiological and clinical associations

DILI represents a major global drug safety concern, with acetaminophen (APAP) overdose remaining one of the most common causes of acute liver failure. In the United States alone, APAP misuse accounts for approximately 500 deaths annually, in addition to nearly 100,000 poisoning-related emergency calls, 50,000 emergency department visits, and 10,000 hospitalizations (67).

APAP is primarily metabolized in the liver by cytochrome P450 enzymes, particularly cytochrome P4502E1 (CYP2E1), to the highly reactive intermediate N-acetyl-p-benzoquinone imine (NAPQI). Excessive NAPQI formation leads to depletion of glutathione (GSH), mitochondrial dysfunction, oxidative stress, and hepatocyte death via ATP depletion, c-Jun N-terminal kinase (JNK) pathway activation, and endoplasmic reticulum stress (68, 69).

Although disturbances in magnesium homeostasis have been increasingly reported in experimental models of APAP-induced hepatotoxicity, systematic clinical data linking magnesium status to DILI risk, severity, or outcomes in humans remain limited.

#### 4.4.2 DILI-specific pathogenic mechanisms

Accumulating experimental evidence suggests that dysregulation of intracellular magnesium homeostasis contributes to APAP-induced hepatocellular injury. In these preclinical systems, APAP exposure leads to a rapid decline in intracellular Mg^2+^, accompanied by reduced ATP production and increased susceptibility to stress (69, 70).

This magnesium loss is mechanistically linked to upregulation of the magnesium efflux transporter CNNM4, observed in both animal models and liver tissues from APAP overdose patients (6). CNNM4 upregulation promotes Mg^2+^ efflux from cytosol and mitochondria, a process exacerbated by mitochondrial membrane depolarization (71). Silencing CNNM4 in experimental settings restores magnesium levels and attenuates injury markers (6).

However, these insights are almost exclusively derived from acute, high-dose APAP models. A critical unanswered question is whether CNNM4-mediated magnesium efflux represents a pathophysiological mechanism common to other forms of DILI, which exhibit vastly different etiologies (e.g., idiosyncratic, immune-mediated, or chronic). This fundamental uncertainty severely limits the current extrapolation of these findings and underscores that CNNM4 remains a preclinical, model-specific target whose broader clinical relevance is, as of now, hypothetical.

#### 4.4.3 Therapeutic interventions and limitations

Preclinical studies in animal models of APAP overdose suggest that magnesium-based interventions may mitigate liver injury through multiple pathways. These include modulating gut microbiota-derived metabolites to suppress the formation of the toxic metabolite NAPQI (72), as well as enhancing mitochondrial biogenesis and function with compounds like MgIG (73).

However, the translational relevance of these findings to human DILI is highly uncertain. The evidence is confined to acute, high-dose APAP models with short observation periods. Clinical data on magnesium supplementation in DILI are sparse, and critically, it is unknown whether modulating magnesium homeostasis can improve hard clinical outcomes—such as progression to acute liver failure or mortality—especially for the many DILI subtypes that are not mediated by APAP-like metabolic activation.

Therefore, while targeting magnesium homeostasis is a plausible adjunctive strategy specifically within the context of APAP toxicity, its general applicability across the etiologically diverse landscape of DILI remains highly speculative. Defining its role will require human studies that move beyond the APAP paradigm to include other common and clinically challenging forms of drug-induced liver injury.

### 4.5 Magnesium deficiency in HCC

#### 4.5.1 Epidemiological and clinical associations

HCC is the sixth most common malignancy and the third leading cause of cancer-related mortality worldwide (74), with a continuously rising global burden. It is projected that HCC-related deaths will increase by approximately 56% by 2040 compared with 2020, reaching nearly 1.3 million annually (75). Due to its insidious clinical onset, most patients are diagnosed at advanced stages, resulting in high recurrence rates and poor prognosis, with a five-year survival rate of less than 53% (76). Although systemic therapies, including immune checkpoint inhibitors (ICIs), have improved outcomes in selected patients (77, 78), the immunosuppressive tumor microenvironment (TME)—characterized by hypoxia, acidity, and oxidative stress—remains a major driver of therapeutic resistance and disease progression (74).

Beyond tumor-intrinsic factors, accumulating epidemiological evidence suggests that systemic magnesium status may be associated with HCC risk. An Italian observational study reported significantly lower serum magnesium concentrations in cirrhotic patients with concomitant HCC compared with those without malignancy (79). Similarly, data from the Mass General Brigham Biobank demonstrated that individuals with hypomagnesemia (serum magnesium <1.70 mg/dL) exhibited a significantly increased risk of incident HCC during follow-up (adjusted HR = 1.88; 95% CI: 1.10–3.22) (80). Consistently, the NIH–AARP Diet and Health Study reported an inverse association between dietary magnesium intake and liver cancer incidence (81).

However, these observational data cannot disentangle causality from confounding or reverse causation. Crucially, the presence of cirrhosis—the dominant risk factor for HCC—itself profoundly disrupts magnesium homeostasis through mechanisms such as malnutrition, portal hypertension, and diuretic use (82). Therefore, low serum magnesium in HCC patients may be largely a consequence of advanced liver disease rather than an independent etiological driver. While these findings robustly establish an epidemiological link, they do not demonstrate that magnesium deficiency initiates hepatocarcinogenesis. Instead, they position magnesium status as a potential biomarker of liver disease severity or a modifiable factor within the pro-carcinogenic milieu of chronic liver injury.

#### 4.5.2 HCC-specific pathogenic mechanisms

Within the context of pre-existing chronic liver disease—the essential precursor to most HCC—mechanistic studies suggest that magnesium deficiency may exacerbate hepatocarcinogenesis by impairing two critical cancer-defensive systems: genomic stability and anti-tumor immunity.

Magnesium deficiency may promote genomic instability. Magnesium is an essential cofactor for DNA repair enzymes. Its deficiency impairs multiple repair pathways—including nucleotide excision repair (NER), base excision repair (BER), mismatch repair (MMR) —thereby compromising genomic instability (83, 84). In HCC pathogenesis, this likely does not constitute the initial oncogenic event but may lower the genomic fidelity of hepatocytes, facilitating the accumulation of mutations and accelerating the evolution of initiated clones within the chronically injured liver.

It also contributes to dysregulation of anti-tumor immunity. Magnesium deficiency fosters a pro-tumorigenic microenvironment by promoting the release of cytokines like TNF-*α*, IL-1, and IL-6 (85). More critically, Mg^2+^ is required for the activation of CD8^+^ T cells via stabilization of LFA-1 (86). Thus, magnesium deficiency could further cripple anti-tumor immune surveillance, which is already severely compromised in the immunosuppressive HCC tumor microenvironment.

However, direct human evidence linking these mechanisms to HCC remains speculative. Studies in non-cancer populations show magnesium modulates T-cell cytokine profiles (87), but whether this translates to meaningful immune control of HCC in patients is unknown.

Collectively, the evidence supports a model wherein magnesium deficiency, often a sequel of advanced liver disease, acts as a disease-modifying amplifier within the established carcinogenic cascade of HCC—worsening genomic integrity and weakening immune control—rather than serving as a primary driver.

#### 4.5.3 Therapeutic interventions and limitations

Growing mechanistic insights have stimulated interest in targeting magnesium homeostasis as a potential adjunctive strategy in HCC. Current experimental approaches are bifurcated: some explore magnesium-based biomaterials (e.g., biodegradable stents) that exert local anti-tumor effects via magnesium ion release in model systems (88); others highlight dysregulated magnesium transporters (e.g., SLC41A1 upregulation) as potential prognostic biomarkers reflecting aggressive tumor biology (89, 90).

However, the translational path for these approaches is fraught with fundamental uncertainties. Direct clinical evidence supporting magnesium-centric interventions for HCC is lacking. Critically, the ubiquitous magnesium deficiency observed in HCC patients is often a consequence of advanced liver disease and cancer cachexia, obscuring its potential role as a therapeutic target. Moreover, key parameters—optimal timing, dosage, and formulation for oncology applications—remain entirely undefined.

Consequently, targeting magnesium homeostasis in HCC must currently be viewed as a purely experimental concept. While preclinical data plausibly link magnesium dysregulation to genomic instability and immune dysfunction, its status as a driver of HCC progression versus an epiphenomenon of late-stage disease is unresolved. Future research must first clarify this causal ambiguity and identify responsive patient subsets before efficacy can be rigorously tested in clinical trials.

## 5 Targeting magnesium homeostasis as a therapeutic strategy for liver diseases

Magnesium deficiency, prevalent in modern diets and often falling below recommended intake levels (310–420 mg/day) (3), is closely linked to liver disease progression. Consequently, restoring magnesium homeostasis is increasingly explored as a potential adjunctive therapeutic approach, supported by clinical studies highlighting its hepatoprotective potential (74, 91, 92).

**Figure 3:**
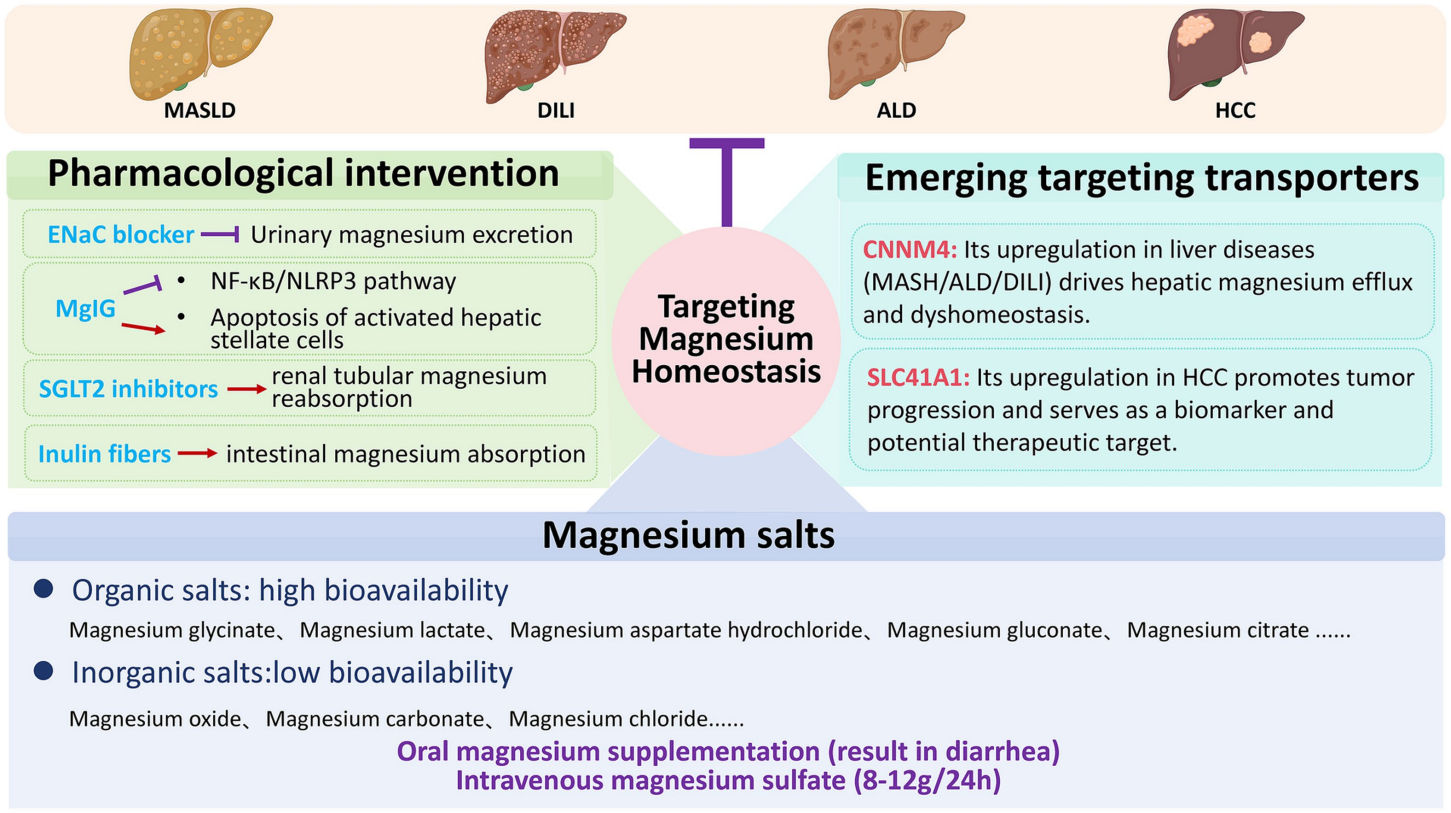
Regulation of magnesium homeostasis through magnesium supplementation, pharmacological intervention, and targeted magnesium transporter modulation inhibits the progression of various liver diseases. MASLD, metabolic dysfunction-associated steatotic liver disease; ALD, alcoholic liver disease; DILI, drug-induced liver injury; HCC, hepatocellular carcinoma; SGLT2, sodium-dependent glucose transporters 2; CNNM4, cyclin M4; SLC41A1, solute carrier family 41 member 1.

This has spurred the development of strategies that operate at multiple levels, from systemic repletion to cellular-targeted interventions. This section reviews these current strategies for restoring magnesium balance in liver diseases. Figure 3 schematically organizes this landscape, spanning general repletion, adjunctive pharmacological modulation, and exploratory transporter-targeted therapies. Crucially, the figure distinguishes clinically applied approaches from preclinical targets, providing a conceptual framework for stratified, mechanism-informed therapy.

### 5.1 Magnesium supplementation: forms, efficacy, and limitations

The primary treatment for hypomagnesemia is magnesium supplementation. Available magnesium formulations vary substantially in absorption rates: organic salts such as magnesium citrate, magnesium aspartate, magnesium glycinate, magnesium gluconate, and magnesium lactate show higher bioavailability compared to inorganic salts like magnesium chloride, magnesium carbonate, and magnesium oxide (93). The magnesium content varies among different oral preparations; specific types, concentrations, and common dosages are detailed in review articles (3). However, oral magnesium supplementation often causes diarrhea (11), limiting its application. In such cases, intravenous magnesium administration may offer better tolerance and efficacy, typically using 8–12 grams of magnesium sulfate within the first 24 h, followed by 4–6 grams daily for 3 to 4 days (94). This short-term intravenous regimen is useful for acute correction of severe hypomagnesemia, but is not suitable for long-term management of chronic liver diseases due to practical constraints and the risk of hypermagnesemia, particularly in patients with coexisting renal impairment.

### 5.2 Evidence from clinical and preclinical studies

Clinical and preclinical studies underscore the biological and therapeutic plausibility of modulating magnesium homeostasis in liver diseases. Evidence from human cohorts suggests benefits of magnesium repletion: in obese individuals with hypomagnesemia, supplementation significantly lowered serum ALT levels (91); higher lifelong magnesium intake was associated with a reduced risk of developing MASLD in middle age (92); and in HCC, magnesium microsphere-enhanced TACE improved objective response rates compared to conventional regimens (74).

However, the efficacy of magnesium interventions appears context-dependent and is not universal. Recent observational data indicate that increased magnesium intake alone may not mitigate MASLD risk in already deficient populations (4). These discrepant results suggest that therapeutic benefits likely depend on factors such as baseline magnesium status, the predominant pathogenic drivers (e.g., inflammation vs. metabolic dysregulation), and disease stage. This heterogeneity underscores the need for precise patient stratification in future clinical trials.

Beyond elemental magnesium, magnesium-containing compounds such as MgIG exhibit multi-targeted hepatoprotective effects in preclinical models. These include the amelioration of lipid metabolism, enhancement of mitochondrial function, and attenuation of inflammation and fibrosis (51, 95, 96). Mechanistically, these benefits are linked to the inhibition of the NF-κB/NLRP3 inflammasome pathway and the induction of apoptosis in activated hepatic stellate cells (97, 98).

Collectively, these findings illustrate both the potential and the complexity of magnesium-based interventions, highlighting that they are not a one-size-fits-all solution but rather a toolkit whose application must be guided by specific disease and patient characteristics.

### 5.3 Adjunctive pharmacological and transporter-targeted therapies

Beyond direct magnesium supplementation, therapeutic strategies can be categorized into those that systemically modulate magnesium handling using repurposed agents, and those that precisely target dysregulated magnesium transporters intrinsic to liver disease pathology.

Systemic modulation through repurposed pharmacological agents offers a pragmatic approach. This includes agents that act on renal conservation [e.g., the modest effect of ENaC inhibitors like amiloride (21), or the more consistent action of SGLT2 inhibitors (3, 99–101)] and those that enhance intestinal uptake [e.g., the prebiotic fiber inulin (102)]. While these interventions can correct systemic deficiency, their effects are indirect and may not adequately address cell-specific magnesium dysregulation driven by the disease process itself.

In contrast, direct targeting of disease-associated magnesium transporters represents a more mechanism-based, albeit exploratory, strategy. The magnesium efflux transporter CNNM4 is upregulated in models of MASH, ALD, and DILI; its silencing restores intracellular magnesium and attenuates injury (6, 8, 12). Similarly, upregulation of the transporter SLC41A1 in HCC correlates with poor prognosis (89, 90). These findings nominate CNNM4 and SLC41A1 as compelling preclinical targets; however, they remain far from clinical application due to the absence of specific pharmacological inhibitors and unresolved questions regarding target safety and specificity in humans.

In summary, the therapeutic landscape ranges from readily deployable systemic modulators to highly specific but investigational transporter-targeted approaches. The translation of both categories, however, is contingent upon overcoming shared and distinct challenges, which we will now examine.

## 6 Translational challenges and future directions

Translating the promise of magnesium homeostasis modulation into effective therapies for liver disease requires overcoming a sequence of interconnected scientific and clinical barriers. These challenges span from accurately identifying the right patients, to delivering therapy precisely to the liver, to safely engaging novel molecular targets, and finally, to demonstrating efficacy in rigorous trials. Addressing this cascade is essential for moving from biological plausibility to clinical practice.

### 6.1 Diagnostic and biomarker limitations

The fundamental first step—accurately identifying which patients have clinically relevant hepatocellular magnesium deficiency—remains elusive. Current reliance on serum magnesium is inadequate, as it poorly reflects intracellular stores (29). This diagnostic gap leads to patient misclassification and likely contributes to heterogeneous responses in trials. Although experimental tools such as magnesium-sensitive fluorescent probes have been developed for preclinical research and provide proof-of-concept for quantifying intracellular magnesium (103–105), they are not yet applicable for routine clinical diagnosis. Therefore, a top translational priority is the development and validation of non-invasive biomarkers capable of assessing intrahepatic magnesium status in humans. Advances in spectroscopic imaging or the adaptation of molecular sensing technologies for clinical use are critical to bridge this gap.

### 6.2 Formulation and delivery challenges

Even with accurate diagnosis, effective delivery of magnesium to hepatocytes is non-trivial. Conventional oral supplements face bioavailability and tolerability issues (11), while intravenous administration is unsuitable for chronic management. Critically, systemic repletion may fail if disease-specific efflux (e.g., via CNNM4) is active. Therefore, innovative strategies such as liver-targeted nanoparticle carriers or prodrugs are needed to achieve sufficient intracellular magnesium concentrations where they are therapeutically required.

### 6.3 Target engagement: from prevalidation to druggability

The development of inhibitors against dysregulated magnesium transporters (e.g., CNNM4, SLC41A1) faces the quintessential challenge of early drug discovery: bridging the vast leap from compelling preclinical data to a viable, safe drug candidate. Key unanswered questions transcend efficacy: Can hepatocyte-specific inhibition be achieved safely? What are the long-term consequences of modulating fundamental ion transport? Human genetic and multi-omics studies are crucial here to validate targets, identify predictive biomarkers, and define patient subsets in whom these precise interventions are most logically applied.

### 6.4 Trial design: evolving from surrogates to mechanistic clinical proof

Ultimately, overcoming the above barriers must be tested within a new generation of clinical trials. Past studies, limited by small size, short duration, and surrogate endpoints (58, 59), are insufficient. Future trials must be mechanism-informed: they should enroll patients stratified by emerging biomarkers of magnesium status or transporter dysregulation, employ standardized and potentially targeted delivery methods, and co-primary endpoints that combine hard clinical outcomes with mechanistic biomarkers (e.g., transporter expression, mitochondrial function) to establish definitive proof of concept.

## 7 Concluding remarks and future perspectives

This review synthesizes evidence supporting the concept that dysregulated magnesium homeostasis functions not as a primary instigator, but as a critical disease-modifying amplifier within the bidirectional pathophysiology of liver diseases. Magnesium deficiency, both a cause and a consequence of hepatic injury, exacerbates core pathological processes including insulin resistance, mitochondrial dysfunction, and immune dysregulation.

Therapeutic strategies ranging from supplementation to transporter-targeted approaches show preclinical promise and early clinical signals. However, their translation is constrained by fundamental challenges: the lack of reliable biomarkers for intracellular magnesium deficiency, inadequate tissue-specific delivery systems, and the immature druggability and unresolved safety profile of key targets such as CNNM4, particularly given their expression beyond the liver.

To advance the field, future research should prioritize a three-pillar translational framework: (1) Diagnostic precision, through the development of validated, non-invasive biomarkers to identify patients with true intracellular deficiency for mechanism-driven trials; (2) Delivery innovation, via liver-targeted strategies capable of overcoming systemic-to-cellular barriers; and (3) Target validation, leveraging human genetic and multi-omics data to establish the causal relevance and therapeutic feasibility of magnesium transporters, including CNNM4 and SLC41A1, and to define responsive patient subsets.

In conclusion, targeting magnesium homeostasis represents a compelling but still exploratory adjunctive strategy in hepatology. Carefully navigating the path from mechanistic insight to clinical validation will be essential to determine whether strategic modulation of this fundamental nutrient–ion axis can be integrated into future, mechanism-informed approaches for liver disease management.
